# Structural Restoration of an Unruptured Vertebral Artery Dissection Following Autologous Mesenchymal Stem Cell Therapy: A Case Report

**DOI:** 10.1002/ccr3.73284

**Published:** 2026-08-10

**Authors:** Hyungchang Kang, Eung Jae Lee, Moon Hyoung Lee, Hyun Soo Kim

**Affiliations:** ^1^ Dr. Kim's Stem Cell Clinic Seoul Republic of Korea; ^2^ Greenhospital Seoul Republic of Korea; ^3^ R&D Center Pharmicell Co. Ltd. Seongnam Republic of Korea

**Keywords:** autologous mesenchymal stem cells, case report, vascular remodeling, vertebral artery dissection, vessel wall MRI

## Abstract

Vertebral artery dissection (VAD) is a recognized cause of ischemic stroke, and current management relies on antithrombotic therapy that is not specifically directed at structural repair of the arterial wall. We report a man in his mid‐50s who presented with a sudden, severe right occipital headache after a golf swing and had a history of a contralateral left VAD 16 years earlier. High‐resolution vessel wall magnetic resonance imaging and angiography showed an acute, unruptured dissection of the right V4 segment with an intramural hematoma, and serial imaging documented progression of the true‐lumen stenosis before treatment. Because the lesion was progressing and stenting of the intracranial V4 segment carried a high risk of iatrogenic rupture, autologous bone marrow–derived mesenchymal stem cells (MSCs) were administered as a well‐tolerated adjunct to continued antithrombotic therapy, in two systemic infusions at 8 weeks and 10 months after onset. The patient had an uneventful clinical course with no infusion‐related adverse events, and follow‐up imaging at 11 months showed resorption of the intramural hematoma and restoration of a normal‐appearing lumen and wall. Because spontaneous healing of unruptured VAD cannot be excluded, this single, uncontrolled observation cannot establish that MSC therapy caused the recovery; nonetheless, the treatment was well‐tolerated and the favorable course warrants evaluation in adequately controlled studies before any therapeutic role of MSCs in VAD can be defined.

## Key Clinical Message

In an unruptured intracranial vertebral artery dissection unsuitable for stenting because of rupture risk, autologous mesenchymal stem cells were given as a well‐tolerated adjunct to antithrombotic therapy, and the dissection resolved on serial vessel wall MRI. Because spontaneous healing cannot be excluded, controlled studies are needed.

**Abbreviations**
BM‐MSC: bone marrow–derived mesenchymal stem cell
GMP: good manufacturing practice
MDCT: multidetector computed tomography
MRA: magnetic resonance angiography
MRI: magnetic resonance imaging
MSC: mesenchymal stem cell
VAD: vertebral artery dissection

## 1 Introduction

Vertebral artery dissection (VAD) is an important cause of ischemic stroke in young and middle‐aged adults. The standard of care for unruptured VAD primarily involves antithrombotic medication to reduce the risk of secondary thromboembolism [1]. Although this strategy reduces ischemic complications, it is not specifically directed at the healing of the intimal tear or resorption of the intramural hematoma, and a proportion of lesions remain vulnerable to pseudoaneurysm formation or progressive stenosis. The natural history of unruptured VAD is variable: although many lesions improve spontaneously over weeks to months with conservative management, others progress, and when the true lumen becomes severely narrowed the available options are constrained, because stenting of the intracranial segment carries a risk of iatrogenic rupture [1, 2, 3].

Mesenchymal stem cells (MSCs) have been investigated as a potential modality for vascular and tissue repair. In preclinical and early clinical studies, MSCs have been reported to modulate sterile inflammation and to promote angiogenesis through paracrine signaling [4, 5]. Although autologous bone marrow–derived MSC products have been commercialized for indications such as acute myocardial infarction, clinical experience with their use in acute arterial dissection is limited. Here, we report the clinical and radiographic course of a patient with an unruptured right VAD who received autologous MSCs (Hearticellgram) 16 years after a severe contralateral VAD. This case highlights the clinical dilemma of a recurrent, unruptured intracranial vertebral artery dissection that was progressing yet unsuitable for stenting, and it documents the course of the lesion on serial high‐resolution vessel wall imaging.

## 2 Case Presentation

### 2.1 Patient Information and Clinical Findings

A Korean man in his mid‐50s presented to the neurology clinic with a severe, acute‐onset headache. The pain was strictly localized to the occipital region, severe in intensity (Numeric Rating Scale Score 8–9), and disrupted his sleep. His medical history was notable for a left cerebellar infarction resulting from a left vertebral artery dissection in April 2008, from which he had recovered without residual neurological deficits. He denied new posterior circulation symptoms such as dizziness, nausea, or vomiting. With respect to his medication history, he had taken clopidogrel for approximately 1–2 years after the 2008 event before discontinuing it.

### 2.2 Timeline

The chronology of the clinical events, imaging studies, and treatments is summarized in Table 1.

**TABLE 1:** Timeline of clinical events, imaging findings, and treatment. Intervals are expressed as days from symptom onset (December 27, 2024 = Day 0).

Date	Interval from onset	Event
April 2008	16 years before onset	Left vertebral artery dissection with left cerebellar infarction; full neurological recovery; clopidogrel taken for 1–2 years and then discontinued
27 Dec 2024	Day 0	Sudden, severe right occipital headache (Numeric Rating Scale 8–9) after a golf swing, with recent alcohol consumption
30 Dec 2024	Day 3	CT angiography: stenosis of the proximal and mid right V4 segment, suspicious for dissection; extradural origin of the right PICA
7 Jan 2025	Day 11	Admission. Vessel wall MRI and MRA: irregular stenosis with intramural hematoma at the right V4 segment (Figure 1). Four‐vessel digital subtraction angiography: stenosis with aneurysmal dilatation and a pseudolumen beyond the PICA origin; chronic dissecting aneurysm at the left V3 segment. Dual antiplatelet therapy started
13 Jan 2025	Day 17	CT angiography: essentially unchanged from January 7. Stenting deferred; antiplatelet therapy de‐escalated to aspirin 100 mg/day with lansoprazole. Discharged
17 Feb 2025	Day 52	CT angiography: further narrowing of the true lumen compared with January 13 (Figure 2)
21 Feb 2025	Day 56 (8 weeks)	First intravenous infusion of autologous bone marrow–derived MSCs (9.0 × 10^7^ cells in 18 mL)
16 Apr 2025	Day 110 (≈3.5 months)	CT angiography: reduction of the intramural hematoma with re‐expansion of the true lumen (Figure 2)
24 Oct 2025	Day 301 (≈10 months)	Second intravenous infusion of autologous bone marrow–derived MSCs (9.0 × 10^7^ cells in 18 mL)
2 Dec 2025	Day 340 (≈11 months)	Vessel wall MRI and MRA: resolution of the intramural hematoma and luminal stenosis at the right V4 segment; left V3 changes unchanged (Figure 3). Aspirin discontinued

Abbreviations: MRA, magnetic resonance angiography; MRI, magnetic resonance imaging; MSC, mesenchymal stem cell; PICA, posterior inferior cerebellar artery.

### 2.3 Diagnostic Assessment

**FIGURE 1:**
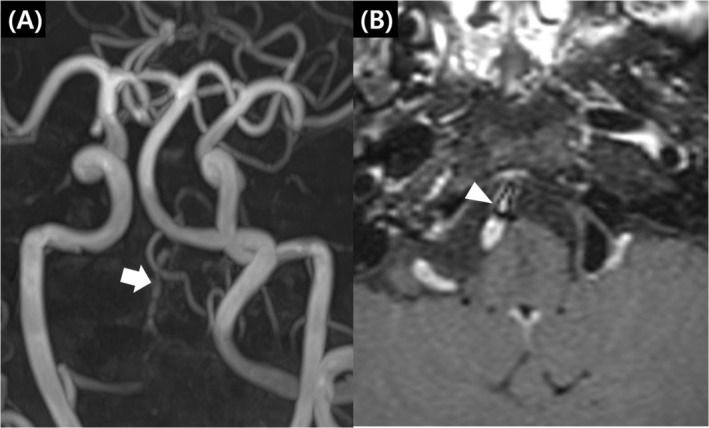
Pretreatment imaging of the acute, unruptured right V4 vertebral artery dissection (January 7, 2025). (A) Frontal maximum intensity projection (MIP) MR angiography (MRA) showing focal narrowing and luminal irregularity of the right V4 segment (arrow). (B) Axial T1‐weighted high‐resolution vessel wall MRI showing a hyperintense intramural hematoma and intimal flap causing eccentric luminal narrowing (arrowhead).

**FIGURE 2:**
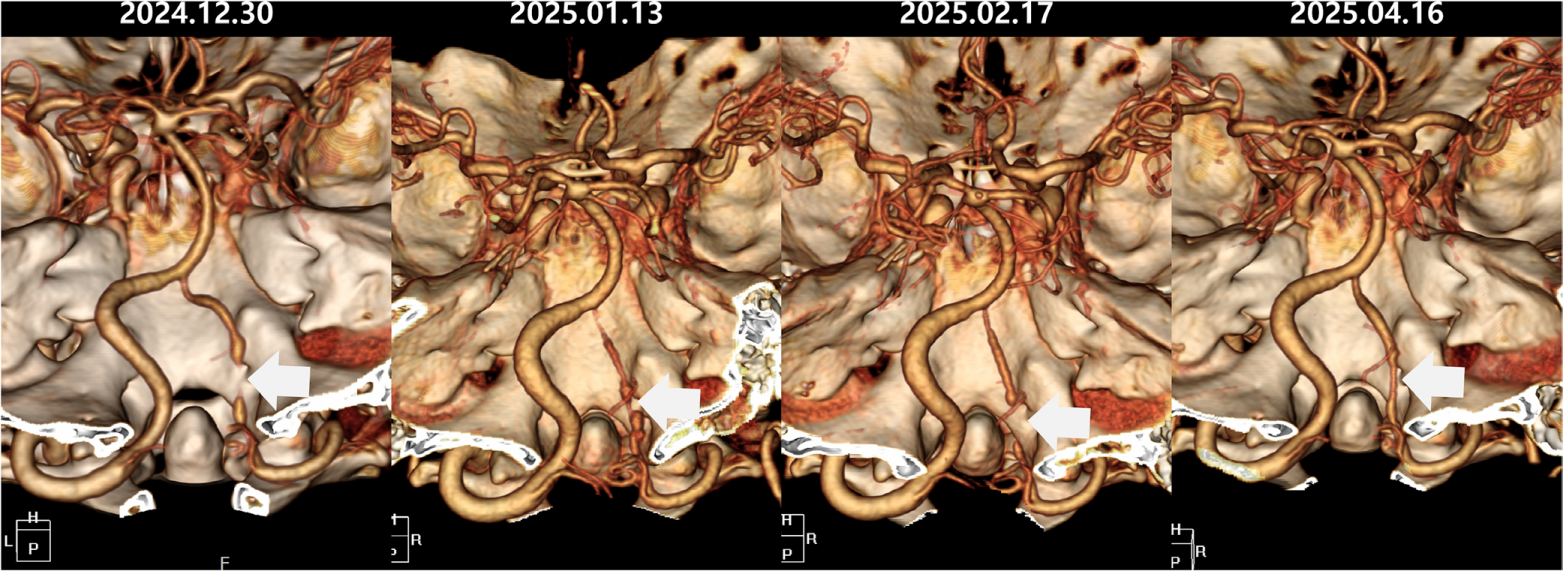
Serial volume‐rendered three‐dimensional CT angiography of the right V4 vertebral artery dissection (arrow). The true lumen was essentially unchanged between December 30, 2024, and January 13, 2025, and had narrowed further by February 17, 2025, without spontaneous resolution; after the first mesenchymal stem cell infusion (February 21, 2025), the April 16, 2025, study shows re‐expansion of the true lumen.

Symptom onset occurred on December 27, 2024, following physical exertion (a golf swing) combined with alcohol consumption. All imaging studies were formally interpreted and reported by the institutional radiology service as part of routine clinical care, and the findings summarized here are drawn from those reports. Initial brain and carotid multidetector computed tomography (MDCT) angiography (December 30, 2024) showed stenosis of the proximal and mid right V4 vertebral artery segment, suspicious for dissection, with an extradural origin of the right posterior inferior cerebellar artery (PICA). Brain MRI and MRA with high‐resolution vessel wall imaging (January 7, 2025) demonstrated irregular stenosis with an intramural hematoma at the right V4 segment, consistent with an acute, unruptured dissection (Figure 1A,B); a small old infarct in the left cerebellar hemisphere and mild leukoaraiosis were also noted. Four‐vessel digital subtraction angiography (January 7, 2025) confirmed abnormal stenosis with aneurysmal dilatation and a pseudolumen at the right V4 segment beyond the PICA origin. On the left, a small, chronic dissecting aneurysm (focal stenosis with partial aneurysmal dilatation) at the V3 segment was attributable to the remote 2008 dissection and remained unchanged on all subsequent studies. The patient was treated with dual antiplatelet therapy; however, because the severely narrowed true lumen and intracranial location placed the segment at risk of rupture and subarachnoid hemorrhage, antiplatelet therapy was de‐escalated to aspirin monotherapy (100 mg/day) with lansoprazole, the risk of bleeding being judged to outweigh the risk of occlusion. Serial CT angiography between December 30, 2024, and February 17, 2025, showed progressive narrowing of the true lumen, most marked on February 17, indicating a lack of early spontaneous resolution (Figure 2); on the source images, the false lumen that had opacified on the earlier studies was no longer apparent by February 17. No dedicated workup for an underlying arteriopathy—including genetic testing, connective tissue evaluation, rheumatologic assessment, or systematic vascular screening—was performed during this episode of care.

### 2.4 Therapeutic Intervention

Stenting of the dissected right V4 segment was considered but deferred: the true lumen was severely narrowed, the intracranial location carried a high risk of iatrogenic rupture and subarachnoid hemorrhage, and the patient was neurologically asymptomatic. With the lesion progressing on serial imaging yet interventional options constrained, antithrombotic therapy was the only established treatment available. In this context, and given the patient's history of a contralateral VAD raising concern for an underlying vascular vulnerability, an autologous bone marrow–derived MSC product (Hearticellgram; Pharmicell Co. Ltd., Seongnam, Republic of Korea) was administered as an adjunctive therapy alongside continued antithrombotic treatment, with the intention of supporting healing of the dissected segment. Its use for vertebral artery dissection was off‐label; the patient gave written informed consent after counseling about this and the absence of established efficacy for the indication.

Hearticellgram (also marketed as Cellgram) is a commercialized autologous bone marrow–derived MSC product that is manufactured under good manufacturing practice (GMP) conditions and is approved by the Korean regulatory authority for acute myocardial infarction [6]. The product was prepared by the manufacturer from the patient's own bone marrow according to its standard manufacturing process.

The patient received two systemic intravenous infusions of cultured autologous MSCs, each comprising 9.0 × 10^7^ cells suspended in 18 mL. The first infusion was administered on February 21, 2025, on Day 56 (8 weeks) after symptom onset and 4 days after the CT angiogram that had documented further narrowing of the true lumen. A second infusion was administered on October 24, 2025, on Day 301 (approximately 10 months) after onset; this was an individual clinical‐care decision made by the treating physicians in view of the patient's underlying predisposition to dissection (the remote contralateral dissection had left a stable, chronic dissecting aneurysm at the left V3 segment), rather than the execution of a predetermined treatment schedule or research protocol, and it was not prompted by clinical deterioration.

### 2.5 Follow‐Up and Outcomes

**FIGURE 3:**
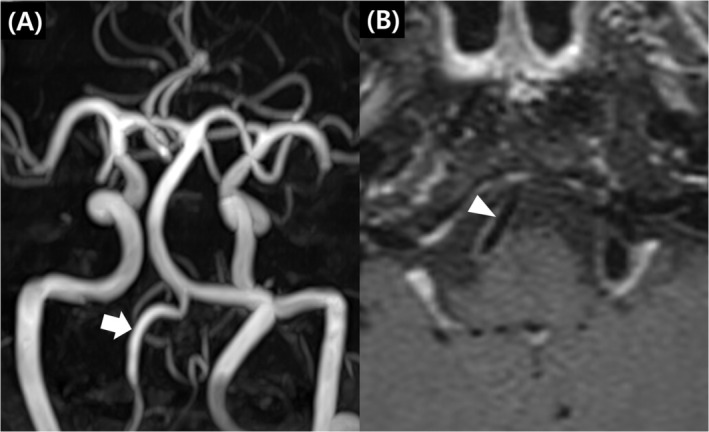
Posttreatment imaging at the 11‐month follow‐up (December 2, 2025). (A) Frontal MIP MRA showing morphological restoration of the right V4 vertebral artery with normal flow, symmetric caliber, and no residual stenosis. (B) Axial T1‐weighted high‐resolution vessel wall MRI showing resorption of the intramural hematoma and normalization of the arterial wall.

Clinically, the patient's severe occipital headache gradually subsided over the course of January 2025 with conservative management. The patient tolerated both intravenous MSC infusions without acute infusion‐related adverse events such as fever, chills, hemodynamic instability, or allergic reaction. An interim CT angiography on April 16, 2025—approximately 8 weeks after the first MSC infusion—showed re‐expansion of the true lumen compared with the February 17 study (Figure 2); the radiology report noted a corresponding decrease in the intramural hematoma on the source images. A subsequent comprehensive follow‐up evaluation on December 2, 2025—approximately 11 months after the initial presentation—included high‐resolution vessel wall MRI and MRA. The previously noted intramural hematoma and luminal stenosis at the right V4 segment had resolved, with restoration of a normal‐appearing lumen and arterial wall (Figure 3A,B), whereas the chronic left V3 changes were unchanged. No new clinical ischemic events, dissecting aneurysms, or hemorrhagic complications were observed during the observation period, and aspirin was discontinued after approximately 11 months of continuous use.

## 3 Discussion

This report describes the radiographic course of an unruptured right V4 VAD in which structural normalization of the artery was observed following systemic autologous MSC therapy administered alongside standard antithrombotic management.

Regarding etiology, the contralateral recurrence after 16 years suggests an underlying predisposition to dissection. Recurrent and bilateral cervical artery dissections raise the possibility of an intrinsic arterial wall vulnerability or an underlying arteriopathy [7]. The acute event was temporally associated with the mechanical torque of a golf swing—which can exert asymmetric shear stress at the V3–V4 junction [8]—together with recent alcohol consumption. The severe occipital headache is consistent with acute distension of adventitial nociceptors by the expanding intramural hematoma, a recognized feature of acute unruptured VAD.

Imaging also showed a small, stable chronic dissecting aneurysm at the left V3 segment, a residual sequela of the remote 2008 dissection. Together with the contralateral recurrence, this suggested a possible systemic tissue vulnerability, and there was clinical concern that reliance on a single patent vertebral artery, or incomplete healing of the new dissection, could pose a long‐term risk to the posterior circulation. Because mechanical interventions carry a high risk of iatrogenic rupture in the intracranial V4 segment—where the absence of an external elastic lamina increases the risk of subarachnoid hemorrhage if the hematoma expands [2]—a noninvasive approach was preferred.

The pretreatment imaging documented a transient progression of stenosis over approximately the first 2 months, followed by morphological improvement after the first MSC infusion. While the improvement coincided with MSC administration, a single observation cannot establish a causal link: the timeline is equally consistent with delayed spontaneous healing, the effect of continued antithrombotic therapy, and the natural evolution of the intramural hematoma, alongside any possible contribution from the MSCs.

The biological mechanisms by which MSCs might contribute to vascular repair have been characterized largely in preclinical models, in which systemically administered MSCs home toward sites of inflammation and tissue injury [9] and secrete paracrine factors and matrix metalloproteinases that may support endothelialization and matrix remodeling [4, 10]. Because intravenously administered MSCs are largely retained in the pulmonary circulation on first pass [11], any systemic effect is more plausibly mediated by these secreted paracrine and immunomodulatory factors than by direct localization of cells to the dissected segment. This mechanism is unproven in the present case: no histopathological, molecular, or biomarker data were obtained, and it therefore remains a hypothesis derived from preclinical data rather than a demonstrated process in this patient.

The first infusion was given on Day 56 (8 weeks), 4 days after imaging had documented further narrowing of the true lumen, and the second on Day 301 (approximately 10 months), both as individual clinical decisions rather than on a predetermined schedule. No data define an interval after the index event within which MSC administration could be expected to act in arterial dissection, and a single case cannot establish one; all that can be said here is that both infusions were given while the lesion remained radiographically unhealed. For the purpose of designing a study, two features of the disease may be relevant. The reparative phase appears to be time‐limited: the signal intensity of the intramural hematoma has been reported to rise over approximately the first 3 weeks and to decline thereafter in lesions that heal [12], and recanalization occurs mainly within the first 6 months [13]. In preclinical models, the paracrine activity attributed to MSCs also depends on the inflammatory signals of actively injured tissue [9]. If MSCs were to act in this setting at all, the acute‐to‐subacute phase would seem the more plausible interval to test. The second infusion, given at 10 months, falls outside that interval; it was directed at the patient's presumed predisposition rather than at the healing lesion.

The contralateral lesion warrants comment in this context. The 2008 dissection had already healed, leaving the stable chronic dissecting aneurysm described above, which is a recognized endpoint of the natural history [3, 14]. A lesion in that state would not be expected to change, and its stability therefore carries no information about the timing or the effect of the infusions.

Whether particular subgroups would be more likely to respond cannot be determined from this case, and no comparative data exist; the following is offered as a consideration for study design rather than as guidance for practice. Most cervical artery dissections lie somewhere between a purely mechanical injury and a defined heritable arteriopathy [7], but the contrast between those poles may still be instructive. Where a dissection follows a mechanical trigger in an otherwise structurally competent wall, the lesion is a discrete injury superimposed on normal matrix, and healing depends on resolution of inflammation, re‐endothelialization, and matrix remodeling, processes that MSC‐derived paracrine factors have been reported to influence after experimental arterial injury [15]. In a heritable connective tissue disorder the position differs, because autologous cells carry the same defect as the vessel. MSCs derived from *FBN1*‐variant pluripotent stem cells in an isogenic model have been reported to produce less fibrillin‐1, to form fragmented microfibrils, and to show increased SMAD2 phosphorylation, abnormalities that resolved when the variant was corrected [16]. If any effect were secretome‐mediated, cells that are themselves abnormal might not reproduce it. This reasoning is untested, and it bears on the present report, since the recurrence after 16 years leaves an underlying arteriopathy unexcluded in this patient and no genetic or connective tissue evaluation was undertaken.

A second axis may be more tractable. Healing propensity can to some extent be gauged on imaging: in one series of 27 unruptured dissections, those whose intramural hematoma signal changed over time healed in every case, against under a quarter of those in which it did not [12]. A lesion with demonstrable reparative activity is the one in which a therapy directed at repair could plausibly act. The same point complicates trial design, since a subgroup that heals reliably without intervention is also the one in which any added effect would be hardest to detect.

With respect to safety, the patient tolerated both intravenous infusions without acute infusion‐related adverse events. A previous report described intrathecal allogeneic stem cell injection for basilar artery dissection [17]. Systemic intravenous administration of MSCs has been reported to be generally well tolerated in clinical studies; a systematic review and meta‐analysis did not identify a significant association with acute infusion‐related toxicity or other serious adverse events, although the available safety data remain limited [18]. In the present case, there were no hemorrhagic or thrombotic complications and no clinical ischemic event.

The natural history of unruptured VAD is generally favorable, and spontaneous morphological improvement is common. In a prospective study of 61 patients with VAD, complete recanalization was observed in 45.9% of patients at 3 months, 62.3% at 6 months, and 63.9% at 12 months, with recanalization occurring mainly within the first 6 months after symptom onset [13]. Earlier serial imaging studies similarly documented progressive luminal improvement over weeks to months, although wall normalization was incomplete in a proportion of patients, who were left with a residual pseudoaneurysm or persistent wall irregularity [3]. The morphological restoration observed in the present case, over an interval of approximately 8–11 months, therefore falls within the timeframe and the range of outcomes expected from the natural history of the disease, and spontaneous healing is an important possibility that cannot be excluded.

The morphological pattern of healing, as distinct from its timing, also varies. High‐resolution vessel wall imaging studies indicate that chronic, spontaneously healed intracranial dissection may resolve along a spectrum: in a series of 29 patients with chronic, unruptured intracranial artery dissection—the great majority involving the vertebral artery—high‐resolution MRI showed complete normalization of both the lumen and the wall in approximately one‐fifth of patients (6 of 29), complete normalization with minimal residual wall change in a further proportion, and incomplete normalization, a dissecting aneurysm, or occlusion in the remainder [14]. These outcomes reflect organization of the intramural hematoma and reparative intimal thickening that characterize the healed dissection [3, 14]; importantly, complete spontaneous normalization, as observed here, is among the recognized outcomes.

The rationale for considering MSCs derives from the larger experience in ischemic stroke, where their paracrine and immunomodulatory properties have been studied. Even in that more extensively investigated setting, however, clinical efficacy is modest and inconsistent—meta‐analyses have reported, at most, small effects on neurological or functional outcomes [19, 20]—and these studies assessed functional recovery rather than structural repair of an arterial wall. No comparable evidence exists for arterial dissection, which underscores the preliminary nature of the present observation.

### 3.1 Limitations

This report has several important limitations. First, as a single, uncontrolled observation, it cannot establish a causal relationship between MSC administration and the observed structural recovery; spontaneous healing, delayed recanalization, and the effect of antithrombotic therapy cannot be excluded. Second, no tissue or biomarker data were obtained, and the imaging findings were assessed qualitatively from the routine clinical reports without quantitative measurement of stenosis, hematoma volume, or wall thickness; the proposed biological mechanisms therefore remain unproven in this patient. Third, the patient experienced two vertebral artery dissections separated by 16 years, which strongly raises the possibility of an underlying arteriopathy such as an inherited connective tissue disorder, fibromuscular dysplasia, or another systemic vasculopathy. However, no genetic testing, connective tissue or rheumatologic evaluation, or comprehensive vascular screening was performed. The absence of such a workup limits the interpretation of both the underlying pathophysiology and the apparent treatment response and represents a significant gap in the characterization of this case. Fourth, the corresponding author and treating physicians have a financial and professional relationship with the manufacturer of the MSC product (see Conflict of Interest Statement); although the radiographic outcomes were objective, this relationship should be considered when interpreting the report. Fifth, follow‐up was limited to approximately 11 months, and the long‐term durability of the observed findings is unknown. Finally, the considerations above regarding the timing of administration and candidate subgroups are extrapolations from preclinical and natural‐history data, not findings of this case.

### 3.2 Patient Perspective

Following the two MSC infusions, the patient returned to his usual daily activities without treatment‐related adverse events. He reported satisfaction with the relief of his initial symptoms and with the radiographic findings at follow‐up.

## 4 Conclusions

In an unruptured right V4 vertebral artery dissection that was progressing on serial imaging and was considered unsuitable for stenting because of the risk of iatrogenic rupture, autologous MSC therapy was administered as a well‐tolerated adjunct to standard antithrombotic treatment, and the dissection subsequently resolved on serial high‐resolution vessel wall imaging without adverse events. Because spontaneous healing of unruptured VAD cannot be excluded and this was a single, uncontrolled observation, a causal contribution of MSC therapy cannot be established. Nonetheless, the favorable anatomical and clinical course and the absence of treatment‐related complications are noteworthy; however, given the uncontrolled design, can only justify adequately controlled studies—incorporating serial vessel wall imaging—to determine whether MSC therapy has any role in VAD, and, if so, at what interval after the index event and in which patients.

## Author Contributions

**Eung Jae Lee:** formal analysis, investigation, writing – review and editing. **Moon Hyoung Lee:** writing – review and editing. **Hyungchang Kang:** conceptualization, data curation, writing – original draft. **Hyun Soo Kim:** supervision, validation, writing – review and editing.

## Funding

This work was funded by Pharmicell Co. Ltd.

## Consent

Written informed consent was obtained from the patient to publish this report and any accompanying images, in accordance with the journal's patient consent policy.

## Conflicts of Interest

H.S.K. is the Chief Executive Officer (CEO) of Pharmicell Co. Ltd., the manufacturer of the autologous mesenchymal stem cell product (Hearticellgram) used in this report. H.K. and M.H.L. are employed as physicians at Dr. Kim's Stem Cell Clinic, which is owned and directed by H.S.K. E.J.L. declares no conflicts of interest. The authors affirm that these affiliations did not influence the clinical care of the patient or the objective acquisition and interpretation of the radiographic data, and the potential for bias is explicitly acknowledged among the limitations above.

## Data Availability

The data supporting the findings of this report are available from the corresponding author upon reasonable request, subject to the protection of patient confidentiality.

## References

[1] S. Debette and D. Leys , “Cervical‐Artery Dissections: Predisposing Factors, Diagnosis, and Outcome,” Lancet Neurology 8, no. 7 (2009): 668–678. 19539238 10.1016/S1474-4422(09)70084-5

[2] I. M. Wilkinson , “The Vertebral Artery: Extracranial and Intracranial Structure,” Archives of Neurology 27, no. 5 (1972): 392–396. 5078895 10.1001/archneur.1972.00490170024004

[3] Y. Yoshimoto and S. Wakai , “Unruptured Intracranial Vertebral Artery Dissection: Clinical Course and Serial Radiographic Imagings,” Stroke 28, no. 2 (1997): 370–374. 9040692 10.1161/01.str.28.2.370

[4] T. Squillaro , G. Peluso , and U. Galderisi , “Clinical Trials With Mesenchymal Stem Cells: An Update,” Cell Transplantation 25, no. 5 (2016): 829–848. 26423725 10.3727/096368915X689622

[5] A. I. Caplan , “Mesenchymal Stem Cells: Time to Change the Name!,” Stem Cells Translational Medicine 6, no. 6 (2017): 1445–1451. 28452204 10.1002/sctm.17-0051 PMC5689741

[6] H. Yang , “South Korea's Stem Cell Approval,” Nature Biotechnology 29, no. 10 (2011): 857–858.

[7] W. I. Schievink , “Spontaneous Dissection of the Carotid and Vertebral Arteries,” New England Journal of Medicine 344, no. 12 (2001): 898–906. 11259724 10.1056/NEJM200103223441206

[8] J. Biller , R. L. Sacco , F. C. Albuquerque , et al., “Cervical Arterial Dissections and Association With Cervical Manipulative Therapy: A Statement for Healthcare Professionals From the American Heart Association/American Stroke Association,” Stroke 45, no. 10 (2014): 3155–3174. 25104849 10.1161/STR.0000000000000016

[9] K. C. Rustad and G. C. Gurtner , “Mesenchymal Stem Cells Home to Sites of Injury and Inflammation,” Advances in Wound Care 1, no. 4 (2012): 147–152. 24527296 10.1089/wound.2011.0314 PMC3623614

[10] O. Honmou , K. Houkin , T. Matsunaga , et al., “Intravenous Administration of Auto Serum‐Expanded Autologous Mesenchymal Stem Cells in Stroke,” Brain 134, no. 6 (2011): 1790–1807. 21493695 10.1093/brain/awr063 PMC3102237

[11] U. M. Fischer , M. T. Harting , F. Jimenez , et al., “Pulmonary Passage Is a Major Obstacle for Intravenous Stem Cell Delivery: The Pulmonary First‐Pass Effect,” Stem Cells and Development 18, no. 5 (2009): 683–692. 19099374 10.1089/scd.2008.0253 PMC3190292

[12] Y. Hashimoto , T. Matsushige , K. Shimonaga , et al., “Monitoring Intramural Hematoma on Vessel Wall Imaging to Evaluate the Healing of Intracranial Vertebral Artery Dissection,” Journal of Stroke and Cerebrovascular Diseases 30, no. 9 (2021): 105992. 34293642 10.1016/j.jstrokecerebrovasdis.2021.105992

[13] A. Arauz , J. M. Márquez , C. Artigas , J. Balderrama , and H. Orrego , “Recanalization of Vertebral Artery Dissection,” Stroke 41, no. 4 (2010): 717–721. 20150549 10.1161/STROKEAHA.109.568790

[14] S. C. Jung , H. S. Kim , C. G. Choi , et al., “Spontaneous and Unruptured Chronic Intracranial Artery Dissection: High‐Resolution Magnetic Resonance Imaging Findings,” Clinical Neuroradiology 28, no. 2 (2018): 171–181. 27677627 10.1007/s00062-016-0544-x

[15] X. Ju , H. Zou , K. Liu , et al., “Meta‐Analysis of the Effect of Mesenchymal Stem Cell Transplantation on Vascular Remodeling After Carotid Balloon Injury in Animal Models,” PLoS One 10, no. 3 (2015): e0120082. 25811171 10.1371/journal.pone.0120082 PMC4374727

[16] J. W. Park , L. Yan , C. Stoddard , et al., “Recapitulating and Correcting Marfan Syndrome in a Cellular Model,” International Journal of Biological Sciences 13, no. 5 (2017): 588–603. 28539832 10.7150/ijbs.19517 PMC5441176

[17] H. Han , S. K. Chang , J. J. Chang , S. H. Hwang , S. H. Han , and B. H. Chun , “Intrathecal Injection of Human Umbilical Cord Blood‐Derived Mesenchymal Stem Cells for the Treatment of Basilar Artery Dissection: A Case Report,” Journal of Medical Case Reports 5, no. 1 (2011): 562. 22136528 10.1186/1752-1947-5-562 PMC3240835

[18] M. M. Lalu , L. McIntyre , C. Pugliese , et al., “Safety of Cell Therapy With Mesenchymal Stromal Cells (Safecell): A Systematic Review and Meta‐Analysis of Clinical Trials,” PLoS One 7, no. 10 (2012): e47559. 23133515 10.1371/journal.pone.0047559 PMC3485008

[19] H. Huang , J. Zhang , J. Lin , and S. Shi , “Efficacy and Safety of Mesenchymal Stem Cells in Patients With Acute Ischemic Stroke: A Meta‐Analysis,” BMC Neurology 24, no. 1 (2024): 48. 38287288 10.1186/s12883-024-03542-1 PMC10823675

[20] Z. Shen , X. Tang , Y. Zhang , et al., “Efficacy and Safety of Mesenchymal Stem Cell Therapies for Ischemic Stroke: A Systematic Review and Meta‐Analysis,” Stem Cells Translational Medicine 13, no. 9 (2024): 886–897. 39159204 10.1093/stcltm/szae040 PMC11386217

